# The secretome from human-derived mesenchymal stem cells augments the activity of antitumor plant extracts in vitro

**DOI:** 10.1007/s00418-024-02265-1

**Published:** 2024-02-24

**Authors:** J. A. Ramirez, M. C. Jiménez, V. Ospina, B. S. Rivera, S. Fiorentino, A. Barreto, L. M. Restrepo

**Affiliations:** 1 Grupo Ingeniería de Tejidos y Terapías Celulares, Facultad de Medicina, Universidad de Antioquia, Carrera 51 A No 62-42, Medellín, Colombia; 2 Grupo de Inmunobiología y Biología Celular, Facultad de Ciencias, Pontificia Universidad Javeriana, Cra 7 No 40-62, Bogotá, Colombia

**Keywords:** Adipose-derived mesenchymal stem cell, Secretome, Tumor microenvironment, Breast neoplasms, Melanoma, Plant extracts

## Abstract

Cancer is understood as a multifactorial disease that involve multiple cell types and phenotypes in the tumor microenvironment (TME). The components of the TME can interact directly or via soluble factors (cytokines, chemokines, growth factors, extracellular vesicles, etc.). Among the cells composing the TME, mesenchymal stem cells (MSCs) appear as a population with debated properties since it has been seen that they can both promote or attenuate tumor progression. For various authors, the main mechanism of interaction of MSCs is through their secretome, the set of molecules secreted into the extracellular milieu, recruiting, and influencing the behavior of other cells in inflammatory environments where they normally reside, such as wounds and tumors. Natural products have been studied as possible cancer treatments, appealing to synergisms between the molecules in their composition; thus, extracts obtained from *Petiveria alliacea* (Anamu-SC) and *Caesalpinia spinosa* (P2Et) have been produced and studied previously on different models, showing promising results. The effect of plant extracts on the MSC secretome has been poorly studied, especially in the context of the TME. Here, we studied the effect of Anamu-SC and P2Et extracts in the human adipose-derived MSC (hAMSC)–tumor cell interaction as a TME model. We also investigated the influence of the hAMSC secretome, in combination with these natural products, on tumor cell hallmarks such as viability, clonogenicity, and migration. In addition, hAMSC gene expression and protein synthesis were evaluated for some key factors in tumor progression in the presence of the extracts by reverse transcription-quantitative polymerase chain reaction (RT-qPCR) and Multiplex, respectively. It was found that the presence of the hAMSC secretome did not affect the cytotoxic or clonogenicity-reducing activities of the natural extracts on cancer cells, and even this secretome can inhibit the migration of these tumor cells, in addition to the fact that the profile of molecules can be modified by natural products. Overall, our findings demonstrate that hAMSC secretome participation in TME interactions can favor the antitumor activities of natural products.

**Supplementary Information:**

The online version contains supplementary material available at 10.1007/s00418-024-02265-1.

## Introduction

Cancer is one of the main causes of death in the world, with more than 9.9 million victims recorded in 2020 (Sung et al. 2021). Followed by colorectal and prostate cancer, breast and lung cancers have the highest lethality rates (GLOBOCAN 2020). Tumor cells have been characterized for having a high proliferation rate, evading growth suppression mechanisms and the immune system as well as resisting programmed cell death, promoting angiogenesis, migration, metastasis, and reprogramming energy metabolism. These features, driven by the cells themselves or influenced by the surrounding tumor microenvironment (TME), contribute to tumor progression (Hanahan and Weinberg 2000, 2011).

TME comprises the cancer and stromal cells, all embedded within a dynamic extracellular matrix. Through this matrix, these different cell populations interact, either directly or through paracrine signaling (Arneth 2020). TME components significantly influence the tumor progression and its potential elimination, in part due to the composition of the immune infiltrate, which can be either enriched in effector or suppressor cells (da Cunha et al. 2019; Duan et al. 2020).

MSCs are currently known for their immunomodulatory capacity, mainly through their secretome (Praveen Kumar et al. 2019); they can also migrate and home in sites of inflammation, including tumors (Caplan 2008, 2019; Nombela-Arrieta et al. 2011). However, their role in the TME has been shown to be paradoxical, either promoting or preventing tumor progression (Hong et al. 2014). There is growing evidence to suggest that as MSCs establish their niches, influenced by their interactions with surrounding cells, they may shift to a pro- or antitumor phenotype (Berger et al. 2016; Hass 2020).

Due to the multifactorial nature of cancer, plant-based therapies offer an alternative to the required multifactorial approach based on the hypothesis that the different metabolites act synergistically on different molecular targets, potentially achieving tumor eradication (Williamson 2001; Lopes et al. 2017). On this basis, we propose to investigate two extracts: one rich in flavonoids obtained from the plant *Petiveria alliacea* (Anamu-SC), which has been shown to have a cytotoxic effect on leukemic and melanoma tumor lines, and the other obtained from the plant *Caesalpinia spinosa* (P2Et), which stands out for its antioxidant capacity, induction of immunogenic cell death, and decrease in tumor size in vivo (Urueña et al. 2015; Gomez-Cadena et al. 2016; Sandoval et al. 2016; Hernández et al. 2017; Ballesteros-Ramírez et al. 2020; Lasso et al. 2020).

Regarding natural products, the roles that they may play in tissue regeneration processes acting on MSCs has been described (Saud et al. 2019). The use of MSCs for delivering antitumor drugs and for modulating tumor angiogenesis through secreted factors has been proposed (Lan et al. 2021). However, there are no clear studies that investigate the effect of MSCs within the TME for therapeutic purposes. In this study, we aim to investigate the effects of the natural products Anamu-SC and P2Et extracts on the interaction between MSC obtained from human adipose tissue (hAMSC) and human melanoma and breast cancer cell lines (A375 y MCF7). The MSC’s secretome is thought to be responsible of several of the MSC properties; therefore, we decided to also study the effect of the hAMSC-conditioned medium (hAMSC-CM) combined with the natural products evaluating their effects on cancer cells viability, migration, and clonogenicity. We further analyzed the expression of secretome-associated molecules that have been reported to play a role in tumor progression or elimination by RT-PCR and Multiplex assay on hAMSCs.

## Material and methods

### Cell culturing

hAMSCs were obtained from the cells bank of the Tissue Engineering and Cell Therapy laboratory and were characterized by flow cytometry (detailed in the Supplemental Material). Tumor cells lines were provided by the Immunobiology and Cell Biology group of Pontificia Universidad Javeriana. Cells were kept in standard conditions as described in the Supplemental Material.

### Natural products

Natural products were produced using *Caesalpinia spinosa* and *Petiveria alliacea* plant materials from Colombia; the Colombian Environmental Ministry authorized the research by the agreement for Access to Genetic Resources and Derived Products no. 220/2018 (RGE 0287-6).

To obtain P2Et, pods and fruits of *Caesalpinia spinosa* (Molina) Kuntze (divi-divi or tara) were collected in the wild, in the municipality of Villa de Leyva—Province of Ricaurte (Department of Boyacá, Colombia) in the polygon delimited by the following geographic coordinates: between 5°37 and #39;95 and #39; and #39; north and 5°39 and #39;17 and #39; and #39; north; and between 73°32 and #39;19 and #39; and #39; west and 73°34 and #39;63 and #39; and #39. Annual average temperatures were recorded between 14.7 and 27.5 °C in the month of March 2013 and, identified by Luis Carlos Jimenez, from the Colombian National Herbarium (voucher specimen L 523714). P2Et, was standardized and chemically characterized as previously described (Sandoval et al. 2016).

On the other hand, the leaves of *Petiveria alliacea* were collected in Quipile, Cundinamarca, Colombia, at a temperature of 24 °C; the plant material was identified by Antonio Luis Mejía from the National Herbarium of Colombia (voucher number COL 333406). The Anamu-SC extract was obtained through supercritical fluids at Corporación Universitaria Lasallista at 60 °C, 400 bar, flow 30 kg/h, and with 15% ethyl acetate as a cosolvent; the procedure was previously described (Ballesteros-Ramírez et al. 2020).

For each assay, both extracts were diluted in 95% ethanol (Merck, Darmstadt, Germany) obtaining a 25 mg/ml fresh solution and homogenized by means of vortex. Working solutions were stored for a maximum of 1 month at 4 °C.

### hAMSC-CM preparation

hAMSCs were plated on 75 cm^2^ culture flask until 80–90% confluency was reached. Then, four washes with Ringer’s lactate, and a final one with MEM (without phenol red and without supplementation, Corning, Corning, USA), were done to remove any traces of platelet lysate. Cells were then cultured in 5 ml Dulbecco’s modified Eagle medium (DMEM)/F12 supplemented with 1% penicillin/streptomycin and 1% l-glutamine for 24 h. hAMSC-CM was collected, centrifugated at 85 RCF for 10 min, and filtered in 0.22 μm syringe filters; then, it was aliquoted and stored at –20 °C until it was used.

### MTT assay

hAMSCs, A375, and MCF7 cell viability was determined using the MTT assay (Alfa Aesar, Ward Hill, USA) after treatment with Anamu-SC, P2Et, or hAMSC-CM, alone or in combination with natural products. 3 × 10^3^ cells were seeded in 96-well plates and allowed to attach overnight. They were treated with serial dilutions of the natural products ranging from 500 to 7.81 μg/ml for 48 h; afterwards, the medium was discarded, and cells were washed once with Ringer’s lactate and MTT was added (final concentration 0.33 mg/ml) and incubated for 4 h. MTT was discarded, and formazan crystals were solubilized in dimethyl sulfoxide (DMSO, Fisher Scientific, Pittsburgh, USA) to measure the absorbance at 540 nm (Liu et al. 1997) (EPOCH microplate reader, BioTek, Winooski, USA). Absorbance values were normalized to negative controls and Doxorubicin 10 nM served as positive control.

### Indirect coculture and viability by Alamar Blue

hAMSCs and tumor cells were cocultured in a transwell system (Corning, Corning, USA), and tumor cell viability was determined by Alamar Blue (InvitrogenTM, Waltham, USA) to model their interaction within the tumor microenvironment. A total of 19 × 10^3^ hAMSCs were seeded on 0.4 μm pore-size transwell inserts directly on the membrane or embedded in 100 μl of fibrin gel (Apical side), and the same amount of A375 or MCF7 (1:1) was seeded on 24-well plate dishes (Basal side) and allowed to attach overnight. Then, cells were treated (both sides equally) with Anamu-SC or P2Et for 48 h using the corresponding IC50 for tumor cells; 10 nM doxorrubicin was used as a positive control and fresh medium as a negative control. Fibrin gels were prepared following the protocol described in (Gaviria et al. 2020). Cell viability was determined by Alamar Blue as instructed by the manufacturer. Briefly, wells were washed once with Ringer’s lactate, and then 500 μl of fresh medium and 50 μl of Alamar Blue were added to each well and left in incubation for 3 h. After that, 100 μl of each well was transferred to a new 96-well plate to measure the absorbance at 570 and 600 nm (EPOCH microplate reader, BioTek, Winooski, USA).

### Colony forming units assay

The clonogenicity of tumor cells treated with natural products was determined using the corresponding IC50, conditioned media, or a combination. For this purpose, 250 × 10^3^ cells per well were seeded in 6-well plates. The following day, treatments or serum-free medium were added as a positive control for 48 h. Cells were then detached and reseed (1 × 10^3^ cells per well) in triplicate in 6-well plates; they were maintained for 10 days, allowing colonies to form in the control group. Colonies were fixed and stained with 0.5% crystal violet (Merck, Darmstadt, Germany) diluted in 80% methanol (Franken et al. 2006). Colonies were segmented and counted using Fiji.

### Migration assay

Tumor cell migration was assessed by the wound healing assay (Grada et al. 2017). A total of 3 × 10^5^ cells were plated on 24-well plates, forming a confluent monolayer using 2.5% fetal bovine serum (FBS) medium. The next day a scratch was done using a 200-μl micropipette tip, the medium was removed, and all wells were washed once with Ringer’s lactate. Serum-free DMEM/F12 medium with 1% l-glutamine or hAMSC-CM was then added with the respective treatments using sublethal doses of the extracts (IC50/10); doxorubicin 1 nM was used as a control. Two pictures per well were taken at 0, 24, and 48 h. The images were then analyzed using Fiji software to determine the percentage of migration inhibition.

1 $${\text{\% Migration Inhibition}} = \frac{{\text{Measured area}}}{{\text{Initial area}}} \times 100\%$$

### Confocal microscopy

MCF7 cytoskeleton arrangement was observed by confocal microscopy using the same treatments as in the wound healing assay. A total of 20 × 10^3^ cells were seeded on fibronectin-pretreated glass slides (Gibco, Grand Island, USA). The next day, the respective treatments were added in duplicate for 24 h. The cells were washed with blocking solution [phosphate-buffered saline (PBS) and 2% SBF], fixed with 4% paraformaldehyde (Sigma, Burlington, USA) for 10 min and permeabilized with triton X 100 0.1% (ICN Biomedicals, Santa Ana, USA) for 5 min; then, they were labeled with Alexa Fluor™ 594 Phalloidin (2 μl/ml in PBS, InvitrogenTM, Waltham, USA) for actin filaments, and DAPI 600 nM (InvitrogenTM, Waltham, US) as nuclear staining. After each labeling they were washed twice with blocking solution. Finally, they were placed on slides with a drop of Prolong (Molecular Probes, Eugene, USA) and allowed to dry overnight to be observed under the microscope using a FV1000 laser scanning microscope from Olympus (Tokyo, Japan). Cells were visualized using an UPLSAPO 60× NA 1.35 objective and fluorescence emission was obtained using 50 mW diode laser line 405 and multiargon FV5-LAMAR 30 mW 543 nm laser line. A total of 640 × 640 images were obtained using X, Y, Z laser scanning and a z-projection was constructed for each field. The experiment was performed in duplicate, and from each condition, at least three different fields were analyzed; on this occasion, only hAMSC-CM from sample 4 was used.

### RT-qPCR for secretome cytokines expression

Total RNA of hAMSC was extracted using TRIzol LS reagent according to the manufacturer’s instructions (Life Technologies Corporation, InvitrogenTM, Waltham, USA) after 48 h treatment with both natural products at a concentration of 60 μg/ml, and RNA quality and quantity were assessed by NanoDrop spectrophotometry (NanoDrop Technologies, Wilmington, USA). Then, complementary DNA (cDNA) was synthesized with the SuperScript III Reverse Transcriptase (InvitrogenTM, Waltham, USA) following the manufacturer’s protocol. Afterwards, the real time polymerase chain reaction (RT-PCR) was carried out using 600 ng of cDNA, iTaq Universal SYBR Green Supermix (BIORAD, Hercules, USA), and 250 nM forward and reverse primers in a total volume of 20 μl (Supplemental Material Table S2). Reactions were done in duplicates using the QuantStudioTM 3 Real-Time PCR system (InvitrogenTM, Waltham, USA).

## Multiplex

The supernatants of the three hAMSCs were collected for protein quantification using the multiplex assay. A total of 3.5 × 10^3^ hAMSCs per well were seeded on 12-well plates, and after 2 h, the TGF-β group was treated with the purified cytokine (Jiménez et al. 2023) at 5 ng/ml during 24 h. TGF-β was then removed, and the extracts were added at 60 μg/ml (average of all IC50s) for another 24 h; subsequently, all the wells were washed five times and FBS free media was added to produce the hAMSC-CM, which, after 24 h, was collected, centrifuged, and transferred to 1.5-ml tubes to be stored at –80 °C until use. Quantification of proteins was done using the MILLIPLEX Human Cytokine/Chemokine Magnetic Bead Panel (HCYTOMAG-60-10) following the manufacturer instructions. Data were processed using Belysa^®^ (Merck, Darmstadt, Germany).

### Statistics

GraphPad Prism 8 software was used for statistical analyses (Boston, USA). Normality was assessed with the Shapiro–Wilk test, and comparisons were made using the Kruskal–Wallis test with Dunn correction for non-normal distributions or, for normal distributions, Student’s *t*-test with Welch correction, assuming nonhomoscedasticity. For the wound assay, the mixed effects model with related values and multiple comparisons with Tukey correction was used. Significant differences were considered when *p* < 0.05.

## Results

### Natural products and the hAMSC-CM act additively to decrease the viability of tumor lines in contrast to the coculture

**Fig. 1:**
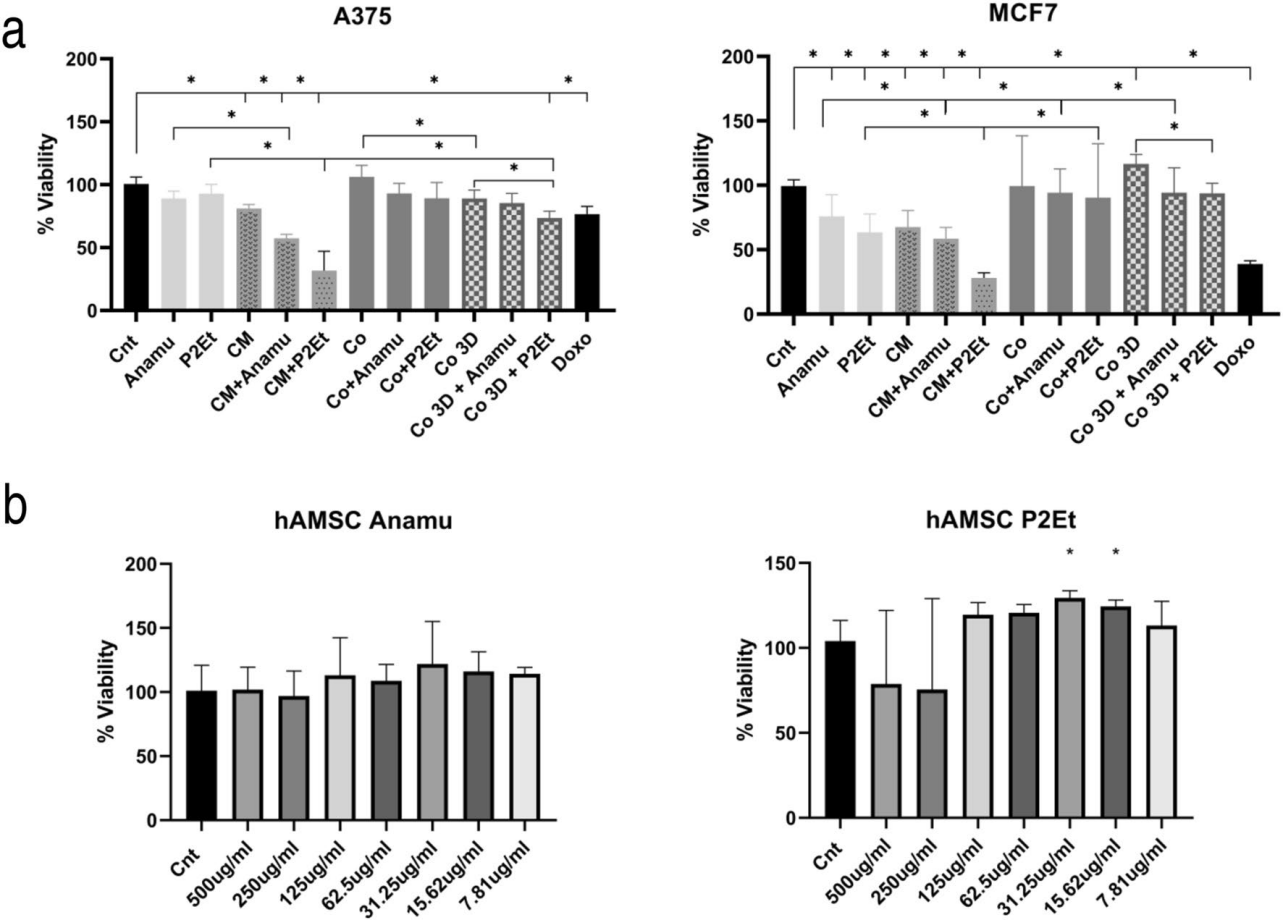
Natural products Anamu-SC and P2Et affect the viability of tumor cell lines and hAMSC. **a** Viability of A375 and MCF7 treated with Anamu-SC, P2Et, hAMSC-CM or combination. Complete media was used as control (Cnt). Tumor cell lines were treated (basolateral zone) with each extract or in indirect coculture (Co) with hAMSC (apical zone) in a 1:1 ratio; hAMSC in fibrin gels were also tested in cocultures (Co 3D). Complete media was used as a negative control (Cnt) and doxorubicine 10 nM (Doxo) as positive control. The median and interquartile range are shown, *N* = 3. **b** Normalized optical density of the hAMSC treated with Anamu-SC and P2Et at different concentrations. P2Et plant extract was obtained from C. spinosa plant pods collected in Villa de Leyva, Boyacá, Colombia and identified by Luis Carlos Jimenez from the Colombian National Herbarium (voucher specimen L 523714). *Petiveria alliacea* leaves were collected in Quipile, Cundinamarca, Colombia and the plant material was identified by Antonio Luis Mejía of the National Herbarium of Colombia (voucher number COL 333406)

The antitumor effect of Anamu-SC and P2Et has been previously proven in several studies (Urueña et al. 2015; Gomez-Cadena et al. 2016; Sandoval et al. 2016; Hernández et al. 2017; Ballesteros-Ramírez et al. 2020; Lasso et al. 2020). Here, we observed a decrease in the viability of breast cancer and melanoma tumor cells in a dose-dependent manner over the course of 48 h of treatment (Supplemental Material Fig. 1a), obtaining the following IC50 values: Anamu-SC on A375 was 79.9 μg/ml, on MCF7 was 41.06 μg/ml, and P2Et on A375 was 61.09 μg/ml and 70.82 μg/ml on MCF7. This effect is less noticeable in A375 when assessed by Alamar Blue using the corresponding IC50s directly, but for MCF7, viability remains decreased by about 50% (Fig. 1a). Treatment with hAMSC-CM decreases the viability of both tumor cell lines evaluated by both techniques, showing a behavior similar to that of the extracts (Fig. 1a and Supplemental Material Fig. 1). Notably, adding either extract to the hAMSC-CM further enhances the viability reduction, particularly with P2Et, evident in both MTT and Alamar Blue assays.

We performed indirect cocultures of hAMSCs and the tumor lines using 0.4-μm transwell systems, facilitating their indirect interaction by seeding the hAMSCs both in two-dimensional culture and in three-dimensional fibrin gels. In this model, only a decrease in viability is significant in the 3DP2Et coculture condition compared with the control; however, the effect is much less pronounced compared with the use of the hAMSC-CM. Moreover, MCF7 viability is significantly increased in three-dimensional coculture, with a similar trend observed in the two-dimensional coculture. Other coculture groups exhibit similar behavior to the control.

To confirm the viability of tumor cells treated with extracts, hAMSC-CM or their combination, the incorporation of propidium iodide was determined as a marker of cell membrane damage. As with MTT and Alamar Blue, a significant decrease in viability was observed in the groups of extracts plus hAMSC-CM at 48 h (Supplemental Material Fig. 1b). Taking together these results, we propose that hAMSC, particularly their secretome, potentiates the cytotoxic effects of the natural extracts.

### hAMSC-CM does not affect the ability of natural extracts to reduce colony formation of A375 cancer cells

**Fig. 2:**
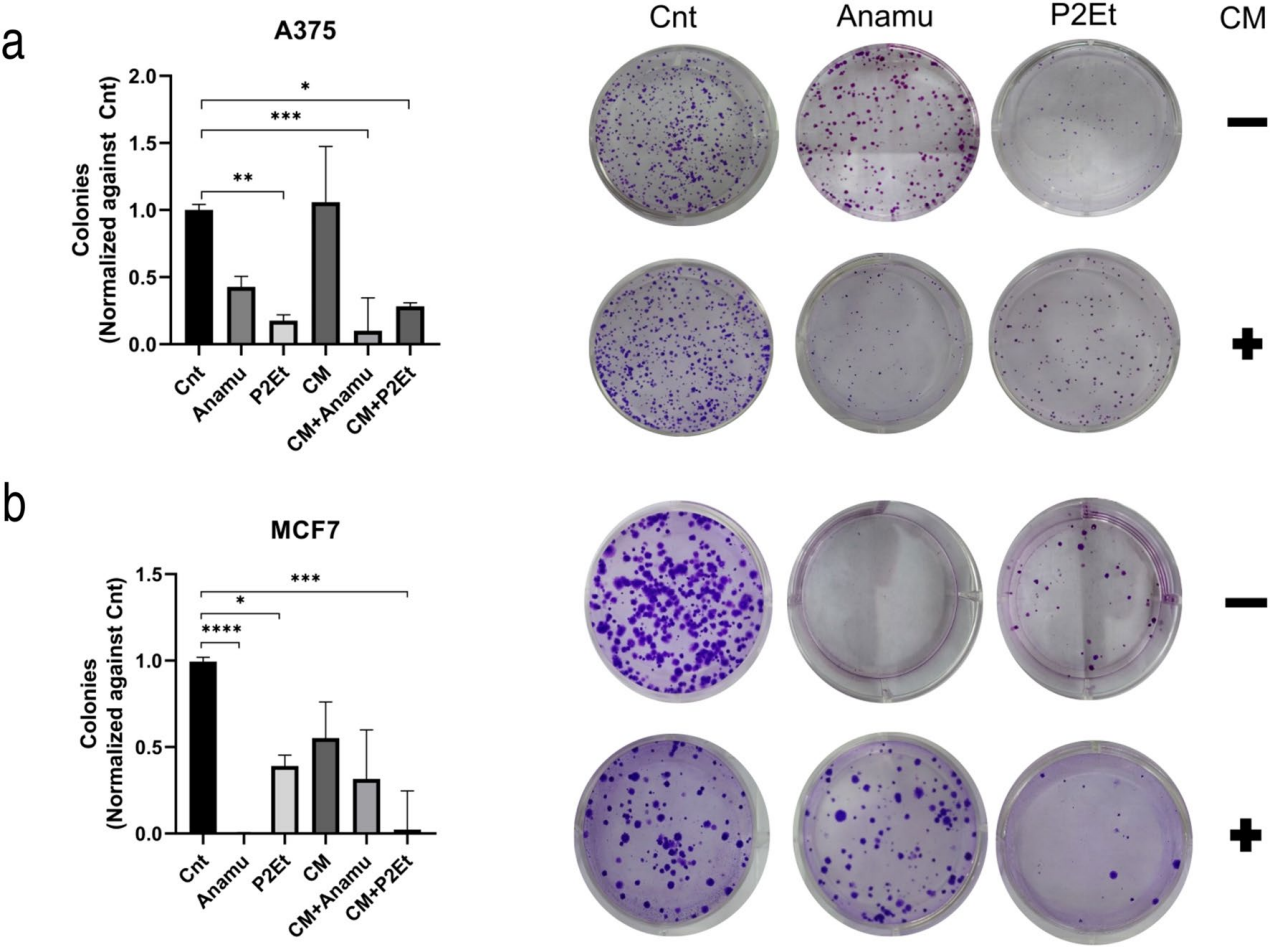
Natural products Anamu-SC and P2Et decrease the clonogenic potential of tumor cell lines. Tumor cell lines A375 (**a**) and MCF7 (**b**) were cultured and treated with the IC50 of each extract, hAMSC-CM or the combination, and then diluted to 1000 cells by well and stained with crystal violet. Medians and interquartile range are shown

The reduction of the colony-forming capacity of different tumor cell lines treated with P2Et has been previously studied (Castañeda et al. 2012; Urueña et al. 2013) but not with Anamu-SC. Here, we investigated whether the hAMSCs could affect the activity of natural products. In this work, this effect is also observed using sublethal concentrations of the extracts (IC50/10) after 48 h of treatment in A375 and MCF7 cells. P2Et significantly reduces the number of both A375 and MCF7 colonies, and Anamu-SC significantly reduces the clonogenic potential of A375 and completely inhibits the formation of MCF7 colonies. On the other hand, hAMSC-CM by itself does not affect colony formation of either A375 or MCF-7, and when the treatment is combined with the natural extracts, it preserves the colony-reducing effect, clearly for A375, although there is a tendency to reverse this effect on MCF-7 (Fig. 2b).

### hAMSC_CM decreases the migration capacity of tumor lines

**Fig. 3:**
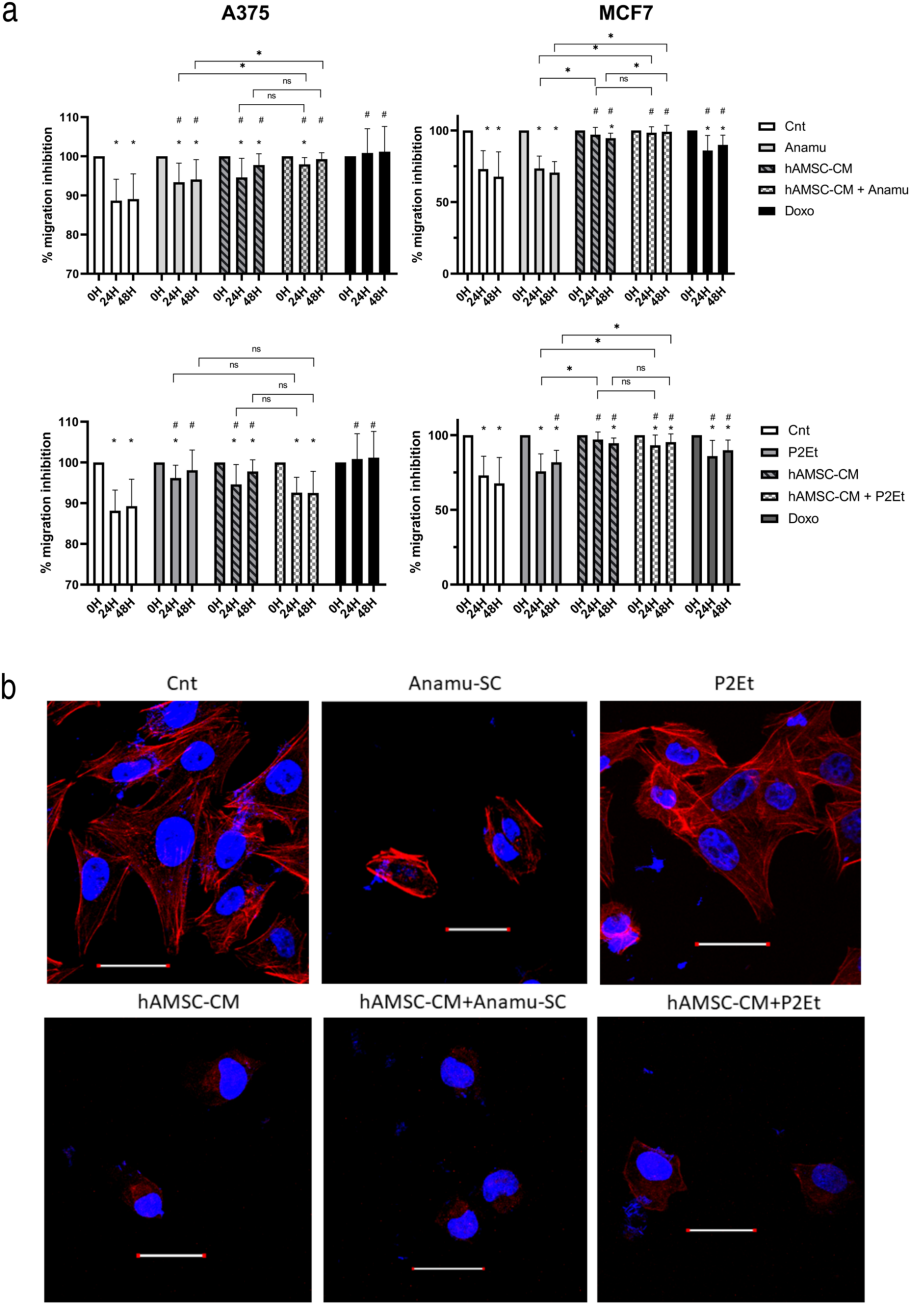
hAMSC-conditioned medium decreases tumor cell lines migration. **a** Individual and combined treatments with hAMSC-CM at 24 and 48 h. Media with standard deviation are shown. Significant differences are considered for values of *p* < 0.05 (* with respect to the value of 0 h and # with respect to the untreated control; differences between treatments are indicated with a bar). Three experiments were performed for each treatment in triplicate and two images were acquired per well (*N* = 3). **b** Representative images of MCF7 cytoskeleton obtained by confocal microscopy (*N* = 2). The experiment was performed in duplicate. From each condition at least three different fields were analyzed, and only hAMSC-CM from sample 4 was used; scale bars are 30 μm

The motility of tumor cells and their ability to migrate collectively was evaluated by wound assay. MCF7 achieved wound closure 22% more than A375 on average while hAMSC-CM significantly decreased the wound closure rate in both tumor lines (Fig. 3a). Additionally, in MCF7, there is no significant difference between the three different times evaluated, suggesting a complete inhibition of collective migration. Both natural products, evaluated at sublethal concentrations (IC50/10), decreased A375 migration but not in MCF7 at 24 h (Fig. 3a). When combining hAMSC-CM with extracts, the effect of the former seems to prevail in all conditions, as no significant differences were found between the hAMSC-CM groups with and without natural products.

Anamu-SC has previously been shown to have the potential to alter the cytoskeleton at the level of actin microfilaments in A375 cells (Urueña et al. 2008). Here, the same effect is observed on MCF7 cells (Fig. 3b); however, these alterations to the cytoskeleton are not observed with P2Et. In agreement with the migration results, hAMSC-CM seems to have the capacity to alter actin microfilaments, and this effect persist when natural products are added, even with P2Et, which by itself does not significantly affect the cytoskeleton.

### P2Et could reverse a protumor-like state in hAMSC

**Fig. 4:**
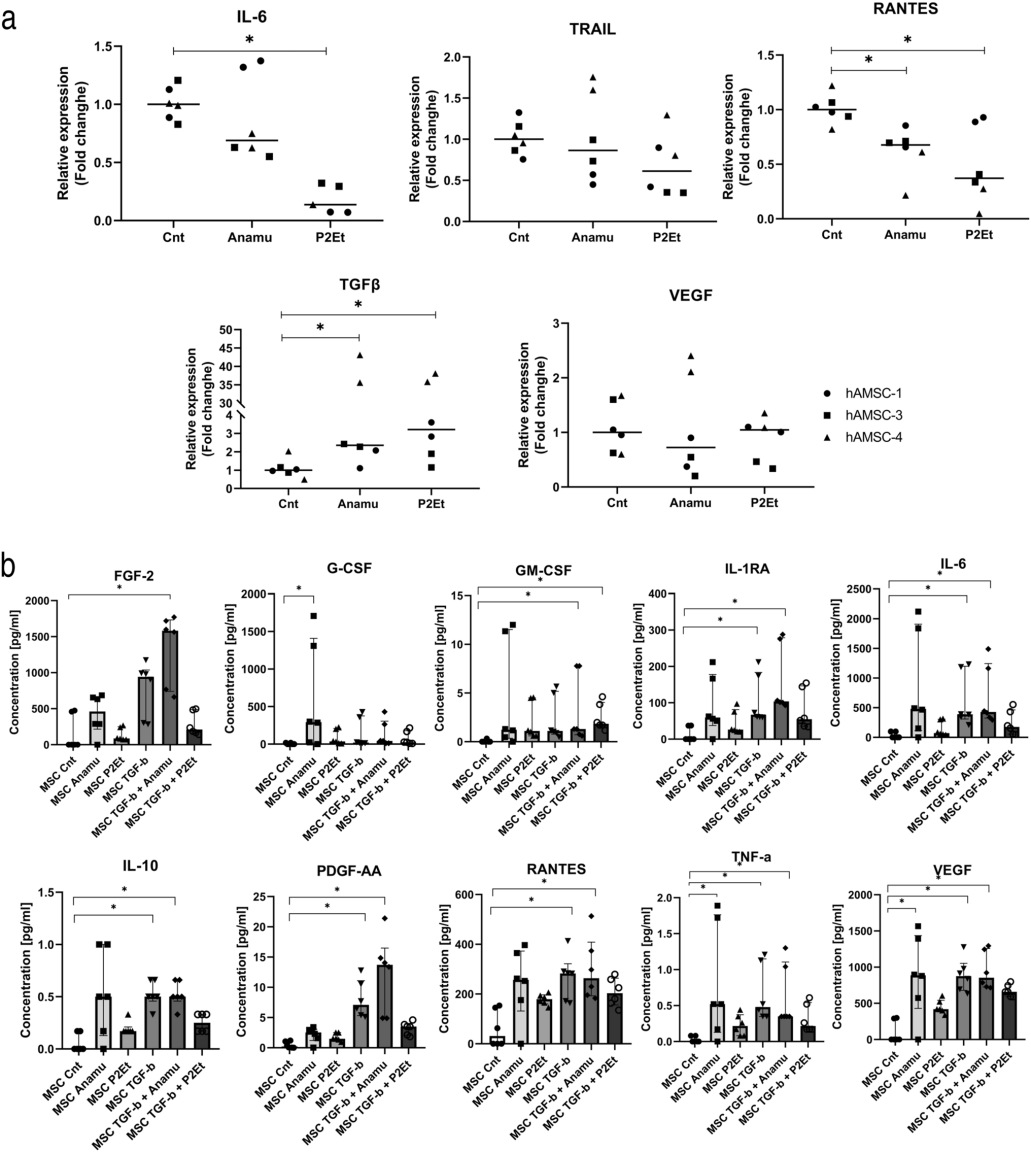
hAMSC treated with Anamu-SC or P2Et (60 µg/ml each) with or without previous activation with TGF-β. TGF-β can increase the synthesis of different factors associated to a protumoral hAMSC phenotype. **a** The 2^−ΔΔCT^ method was used to determine the relative changes in gene expression as compared to the house-keeping β2-microglobulin on hAMSC treated with both natural products for 48 h. Two experiments were performed in duplicate. **b** The HCYTOMAG-60-10 multiplex panel was used to quantify the presence of various factors in the hAMSC-CM, every hAMSC (three in total) sample was tested by duplicate and the data were analyzed using the manufacturer software Belysa^®^; comparisons were made by the Kruskal–Wallis test

Motivated by the hAMSC-CM effects described above, we sought to partially characterize its secretome and assess the effect of natural products on it. Notably, immunomodulation is a core feature of MSCs, as they act as signaling cells secreting trophic factors such as cytokines and growth factors. Natural products’ effect on MSC differentiation and proliferation of MSCs (Saud et al. 2019) has been studied, but little has been studied on their impact on the hAMSC secretome. In this work, we selected some of the cytokines that have been reported to be part of the hAMSC secretome and that also play a role in tumor progression. Figure 4a shows the variation in gene expression in the three different hAMSC samples. It is possible to evidence variations in the response to natural products in the three samples, especially in the expression of TGF-β in hAMSC-4, which is greater than 30-fold with respect to the control versus two- to threefold in the other two samples. P2Et was able to significantly reduce the expression of IL-6, one of the main cytokines present in the tumor microenvironment favoring tumorigenesis and tumor progression(Kumari et al. 2016), and RANTES, known to favor tumor progression and metastasis (Karnoub et al. 2007), while not significant differences were detected in the levels of released IL-6 and RANTES as evaluated by Multiplex (Fig. 4a). The relative expression of TRAIL, a potent inducer of apoptosis, was not significantly modified. Angiogenesis is one of the hallmarks of cancer, favored in hypoxic environments such as the TME and induced by growth factors such as vascular endothelial growth factor (VEGF, Carmeliet 2005). The expression of this growth factor was confirmed in the three hAMSCs; however, no significant differences were found after treatment with natural products, although there was an increase in expression in hAMSC-4 treated with Anamu-SC, which was also increased in the secretome. While P2Et did not significantly change the synthesis of the evaluated factors, hAMSC treated with Anamu-SC showed a significant increase in G-CSF, TNF-α, and VEGF.

TGF-β, known as a potent inducer of a protumor state in different cell types including MSCs and fibroblasts (CAFs), significantly increased the expression and synthesis of FGF-2, IL-1Ra, RANTES, IL-6, IL-10, PDGF-AA, VEGF, TNF-α, or TGF-β itself in hAMSC treated with 5 ng/ml of recombinant TGF-β (Fig. 4 and Supplemental Material Fig. 3). Interestingly, it also a decreased TRAIL expression (Supplemental Material Fig. 3). We investigated the potential of the extracts to revert the protumor state induced by TGF-β after 24 h. The use of Anamu-SC after TGF-β exposure showed no significant difference; however, P2Et showed a decrease expression of FGF-2, IL-6, IL-10, PDGF-AA, and TNF-α (Fig. 4).

## Discussion

The use of medicinal plants is already well known and accepted in multiple scenarios and has been promoted by the WHO (Farnsworth et al. 1985). Currently, there are several published studies where the effect of promoting the proliferation and differentiation of hAMSCs given by some plant derivatives with potential use in regenerative medicine can be evidenced (Khalilpourfarshbafi et al. 2019; Buhrmann et al. 2020). Up to the date of this work, little research has been done on the use of products derived from *Petiveria Alliacea* or *Caesalpinia spinosa* in hAMSC cultures. Reports of the use of products rich in flavonoids such as Naringin, also used as antineoplastic (Memariani et al. 2021), indicate concentration-dependent increases of BM-MSC proliferation through ERK activation and promoting osteogenic differentiation via expression of Runx2, OXS, CON, and Col1 (Wang et al. 2017; Lavrador et al. 2018; Saud et al. 2019). A similar effect was observed with Icariin, a flavonoid glycoside with anticancer properties (Qin et al. 2015). In resveratrol, a polyphenolic natural product with antioxidant, anti-inflammatory and antitumor properties have been used at low concentrations to favor MSC proliferation via activation of the ERK1/2 and MAPK pathway. In addition, it can participate in osteogenic, adipogenic, and neurogenic differentiation; although, in some cases, an inhibitory effect of adipogenic differentiation has been reported (Peltz et al. 2012; Saud et al. 2019; Hu and Li 2019).

While indirect coculture with hAMSCs did not significantly alter tumor cell growth (Fig. 1a), hAMSC-4 increased the viability of tumor cells in some cases. Notably, cocultured tumor cells better withstand treatment with the extracts. In a study by Preisner and collaborators, they investigated the impact of hAMSCs on four melanoma cell lines and two primary melanoma cultures, finding that both tumor cells and hAMSCs acquire a protumoral phenotype favoring migration, invasion, and angiogenesis but few differences in the proliferation of both hAMSCs and melanoma lines (Preisner et al. 2018), similar to what was found in this work. Similarly, Koellensperger et al. studied the interaction of hAMSCs and different breast cancer lines, including MCF7, finding no significant differences in viability, similar to the results of this work, but did find changes in gene expression in both populations compared with monoculture; besides, secretion of MMP-1, MMP-3, MMP-10, HGF, IL-6, IL-8, IL-12, and VEGF by MCF7 was detected, while hAMSCs showed minimal changes, accompanied by an increase in migration of both MCF7 and hAMSCs and an increase in the invasive potential of hAMSCs but not of MCF7 (Koellensperger et al. 2017). As discussed, MSCs are sensitive to the microenvironment, and as such, their phenotype is related to it. In general, MSCs present in the TME acquire a phenotype that supports tumor growth (Berger et al. 2016; Hass 2020) either by the release of trophic factors, as shown in this work (Fig. 4 and Supplemental Material Fig. 3), or differentiation to other cell types such as cancer-associated fibroblasts (Barcellos-de-Souza et al. 2016).

On the other hand, hAMSC-CM, similar to the extracts at their IC50 concentrations, significantly reduced tumor cell viability. Importantly, this effect was additive when hAMSC-CM was combined with extracts, particularly evident with P2Et, and extended to the clonogenic potential of tumor cells (Figs. 1a and 2). When viability was evaluated by propidium iodide (PI) incorporation, it was also visible that the combined treatment significantly reduced the viability of both tumor cell lines after 48 h, showing an additive effect (Supplemental Material Fig. 1b). Other works have shown that MSC-conditioned media could induce immunogenic cell death (Lin et al. 2017), a mechanism previously observed in tumor cells treated with P2Et (Castañeda et al. 2012), and hAMSC-CM cultured at high density for three days triggered induced cell death in MCF7 cells and high expression of INF-β via JAK/STAT1 pathway (Ryu et al. 2014).

Treatment with hAMSC-CM significantly reduced collective cell migration in tumor cells (Fig. 3), aligning with reports from other groups for A375 and MCF7 (Ahn et al. 2015; Visweswaran et al. 2018). Treatment with the extracts individually showed a lower antimigratory effect than the hAMSC-CM group, suggesting that the effect observed in the combined treatment is mainly due to hAMSC-CM. In agreement with what was observed, a study by Clarke et al. showed that conditioned medium obtained from an immortalized MSC line inhibited the migration of MDA-MB-231 breast cancer tumor cells, an effect partially attributed to the expression of TIMP-1/2, which suppresses the activity of MMP-1, MMP-2, and MMP-9 (Clarke et al. 2015). The decrease in MMP-2- and MMP-9-mediated migration has also been demonstrated in A375 and MCF7 lines (Li et al. 2014; Ji et al. 2015) in both cases in NF-κb inhibition. In the same context, it has been observed that MSCs can secrete IL-1Ra by preventing activation of the NF-κB pathway (Volarevic et al. 2010) and was confirmed here when assessed by multiplex (Fig. 4), albeit at low levels.

Cytoskeleton alterations induced by Anamu-SC were previously reported on A375 cells (Urueña et al. 2008). Here, we observed a similar effect in MCF7 cells; additionally, hAMSC-CM seems to also alter the MCF7 cytoskeleton, and this effect is preserved in the combined treatment with Anamu-SC, and P2Et, where the natural products alone do not present this effect. These findings align with the decrease in tumor cell migration (Fig. 3b).

TNF-β altered the secretome profile of hAMSC, increasing the concentration of growth factors and cytokines, such as FGF-2, IL-6, IL-10, PDGF-AA, RANTES ,and VEGF, but also decreased the expression of antitumoral factors such as TRAIL (Fig. 4b and Supplemental Material Fig. 3). Researchers have aimed to revert the protumoral phenotype in different stromal cells within the tumor microenvironment, including MSC; however, limited research exists about the potential use of natural products. A previous work showed that the use of polyphenol-rich compounds can decrease the release of proinflammatory cytokines such as IL-6 or IL-8 in MSCs previously exposed to an acidic environment (Di Pompo et al. 2021); similarly, in our work we found that P2Et can decrease several protumoral factors after treatment with TGF-β. Conversely, Prakoeswa and collegues (Prakoeswa et al. 2020) reported that resveratrol treatment increases MSC production of protumor factors EGF, HGF, PDGF, and TGF-β. In the present work, it was also observed that levels of factors such as FGF-2, IL-6, IL-10, RANTES, or VEGF increased when hAMSC was treated with Anamu-SC, and the use of Anamu-SC after TGF-β stimulation showed minimal impact.

In view of the alterations that have been observed in the phenotype of MSCs due to the direct or paracrine interaction with components of the TME (Kapur and Katz 2013; Maumus et al. 2013; Zimmerlin et al. 2013), this suggests the potential of obtaining trophic factors from MSCs under controlled conditions, without the risk of facing the variability and possible induction of protumor characteristics when exposing the cells to the TME. It was depicted in this work that hAMSC can protect tumor cells from the cytotoxic effect of the natural products when they were in coculture (two dimensional and three dimensional), perhaps by the release of tumor supporting cytokines induced by the crosstalk, but the effect of the conditioned medium showed antitumor activity and worked additively with the plant extracts in decreasing tumor cells viability, clonogenicity, and migration. Currently, researchers are aiming to target different components from the TME as these have shown to play a key role in tumor progression. It is here where P2Et, besides having antitumor properties, also showed promising results on reverting the protumorigenic state of hAMSC in this system.

### Supplementary Information

Below is the link to the electronic supplementary material.

Supplementary file1 (TIFF 26919 KB) Natural products Anamu-SC and P2Et act additively with hAMSC-CM decreasing tumor cell lines viability. a) Tumor cells mortality assessed by MTT increased when treated with the extracts in a dose-dependent manner. hAMSC-CM alone can induce tumor cells death, and the effect could increase when combined with the extracts after 48 hours of treatment as evidenced in b) where viability was assessed by PI incorporation. FBS-free medium was used as a positive control. Median and interquartile range are shown. At least 5000 events were acquired (N=3)

Supplementary file2 (TIFF 18224 KB) Representative images of the migration assay. Individual and combined treatments with hAMSC-CM at 24 and 48 hours

Supplementary file3 (TIFF 9084 KB) TGF-β induces the expression of different genes associated to a pro-tumoral hAMSC phenotype. hAMSC-4 were tested by duplicate and the 2-ΔΔCT method was used to determine the relative changes in gene expression as compared to the House Keeping β2-Microglobulin

Supplementary file4 (DOCX 21 KB)

## Data Availability

No datasets were generated or analyzed during the current study.
