# Supplementary figures and images for: The secretome from human-derived mesenchymal stem cells augments the activity of antitumor plant extracts in vitro
Source: Histochem Cell Biol. 2024 Feb 24;161(5):409–21. doi: 10.1007/s00418-024-02265-1 (PMC11045572; doi:10.1007/s00418-024-02265-1)

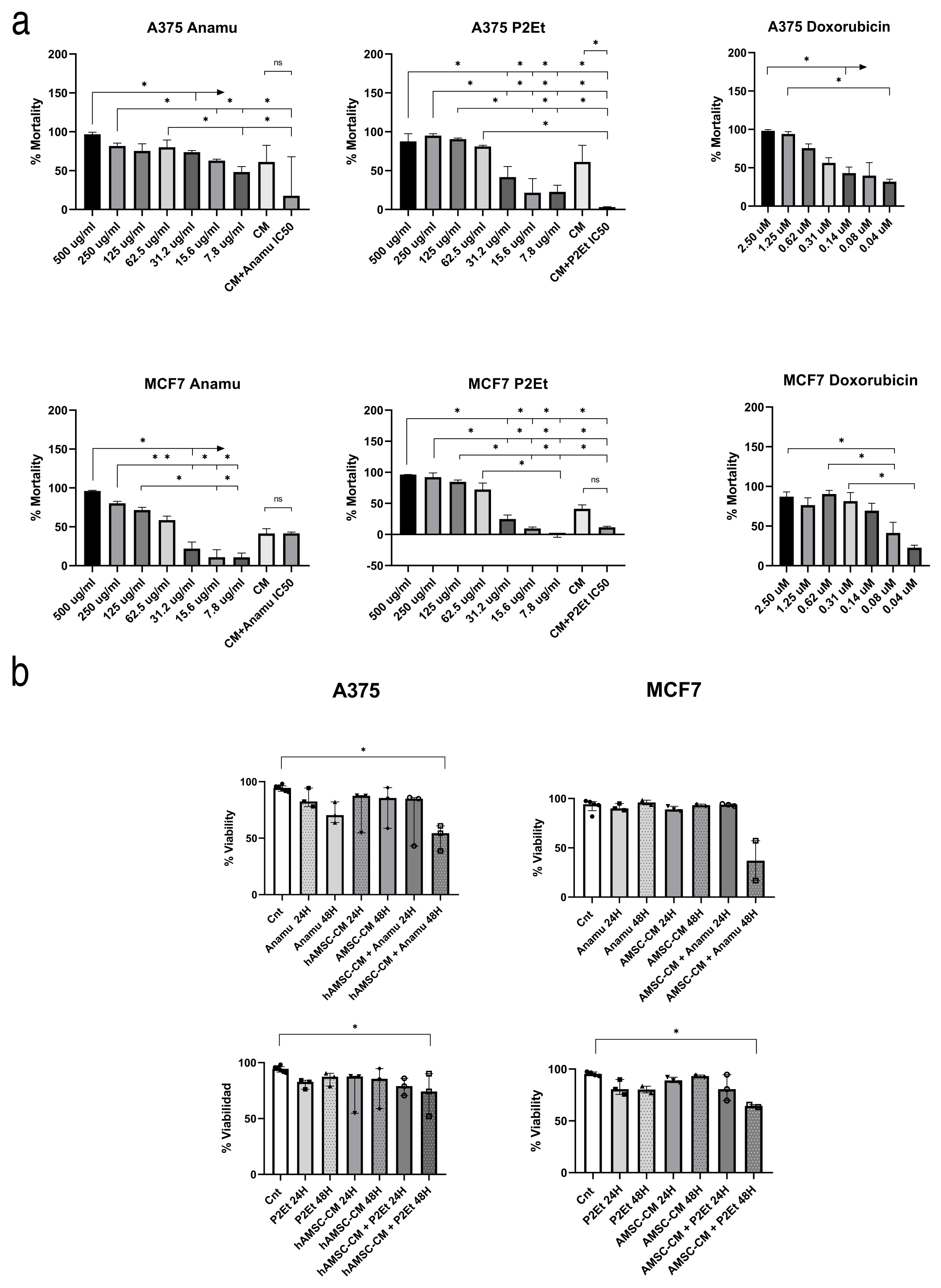

Supplement: Supplementary file 1 — Supplementary file1 (TIFF 26919 KB) Natural products Anamu-SC and P2Et act additively with hAMSC-CM decreasing tumor cell lines viability. a) Tumor cells mortality assessed by MTT increased when treated with the extracts in a dose-dependent manner. hAMSC-CM alone can induce tumor cells death, and the effect could increase when combined with the extracts after 48 hours of treatment as evidenced in b) where viability was assessed by PI incorporation. FBS-free medium was used as a positive control. Median and interquartile range are shown. At least 5000 events were acquired (N=3) [file 418_2024_2265_MOESM1_ESM.tiff]

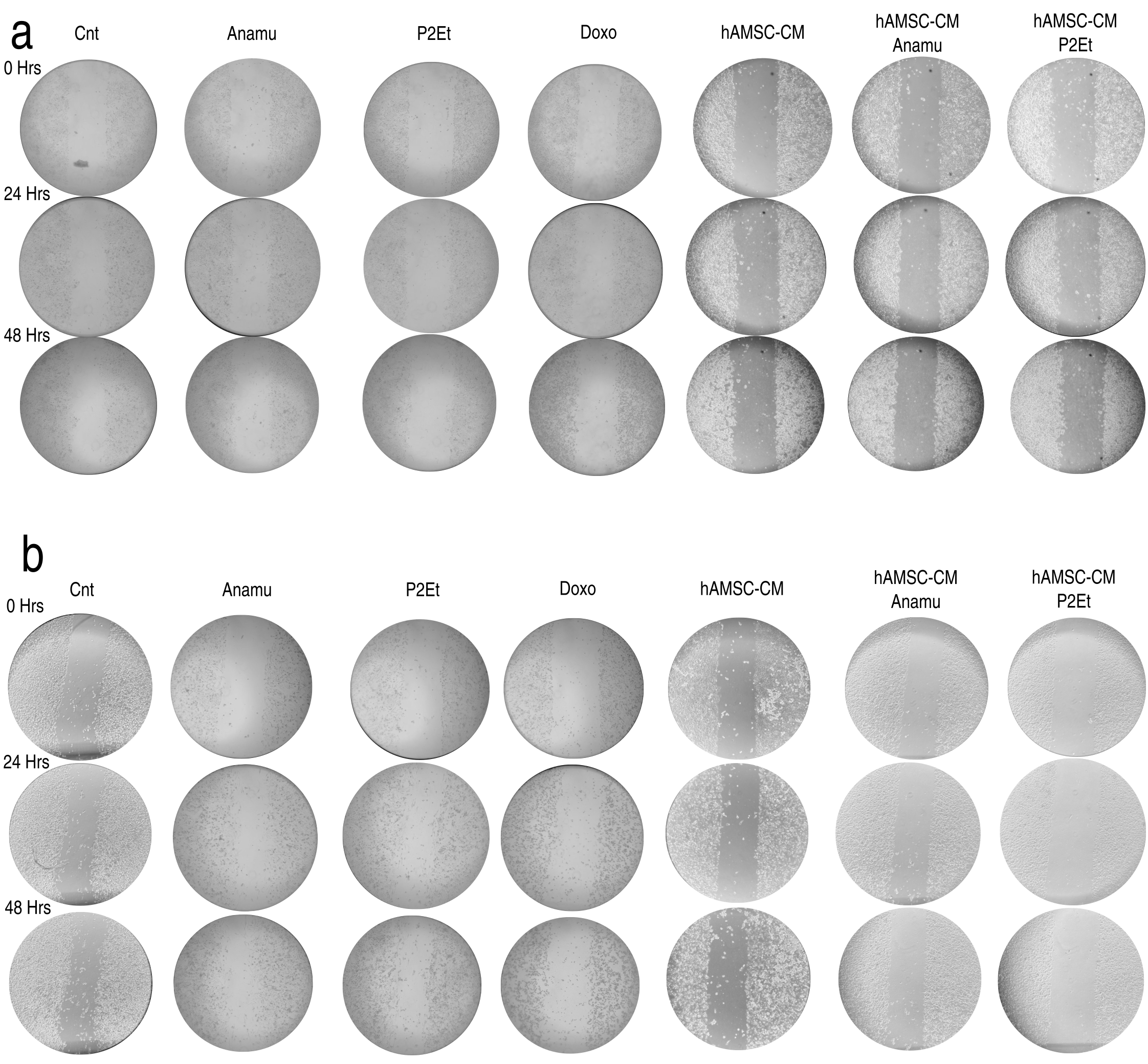

Supplement: Supplementary file 2 — Supplementary file2 (TIFF 18224 KB) Representative images of the migration assay. Individual and combined treatments with hAMSC-CM at 24 and 48 hours [file 418_2024_2265_MOESM2_ESM.tiff]

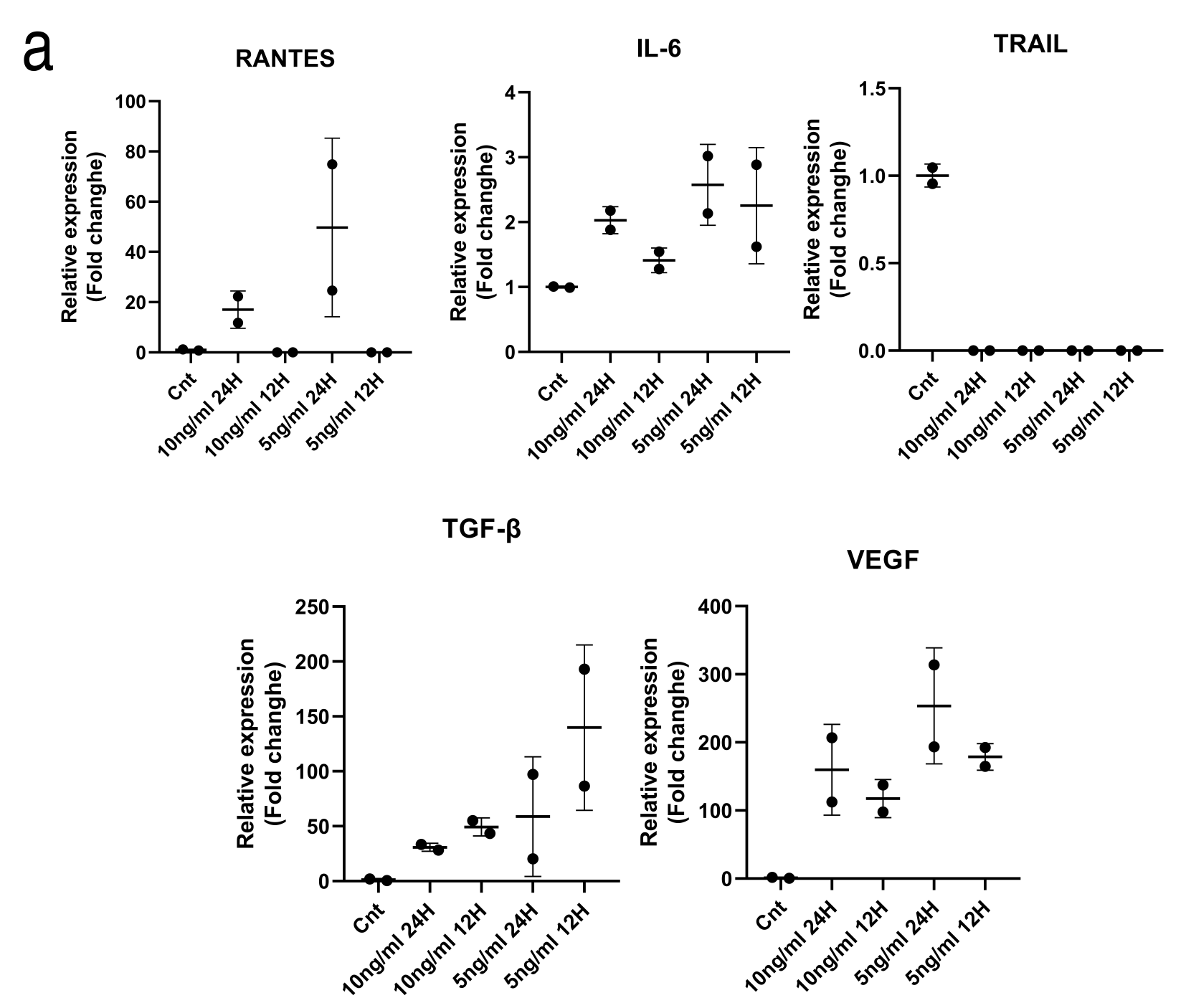

Supplement: Supplementary file 3 — Supplementary file3 (TIFF 9084 KB) TGF-β induces the expression of different genes associated to a pro-tumoral hAMSC phenotype. hAMSC-4 were tested by duplicate and the 2-ΔΔCT method was used to determine the relative changes in gene expression as compared to the House Keeping β2-Microglobulin [file 418_2024_2265_MOESM3_ESM.tiff]
