# Supplementary material for: The secretome from human-derived mesenchymal stem cells augments the activity of antitumor plant extracts in vitro
Source: Histochem Cell Biol. 2024 Feb 24;161(5):409–21. doi: 10.1007/s00418-024-02265-1 (PMC11045572; doi:10.1007/s00418-024-02265-1)
Supplement: Supplementary file 4 — Supplementary file4 (DOCX 21 KB) [file 418_2024_2265_MOESM4_ESM.docx]

**Supplementary information**

**The secretome from human-derived mesenchymal stem cells augments the activity of antitumor plant extracts *in vitro***

**Journal:** Histochemistry and Cell Biology.

J A. Ramirez^1^, M C. Jiménez^2^, V. Ospina^1^, B S. Rivera^1^, S. Fiorentino^2^, A. Barreto^2^, L M. Restrepo^1^.

^1^Grupo Ingeniería de Tejidos y Terapías Celulares, Facultad de Medicina, Universidad de Antioquia, Carrera 51 A No 62-42, Medellín, Colombia.

^2^Grupo de Inmunobiología y biología celular, Facultad de Ciencias, Pontificia Universidad Javeriana, Cra 7 No 40 – 62, Bogotá, Colombia.

*Corresponding author:

DSc. Luz Marina Restrepo.

Email: [marina.restrepo@udea.edu.co](mailto:marina.restrepo@udea.edu.co)

**Supplementary methods:**

**Human-derived Adipose Mesenchymal Stem Cells (hAMSC) isolation:**

The human cells used in this work belong to the Tissue Engineering and Cell Therapy lab’s cells bank (UdeA, Medellin). After informed consent, hAMSC from three different donors were isolated from the vascular stromal fraction as.

**Cell culturing:**

A375 and MCF7 cell lines were provided by the Immunobiology and Cell Biology group of Pontificia Universidad Javeriana. Tumor cell lines were cultured using DMEM/F12 (Eurobio, Essonnes, France) media supplemented with 5% FBS (Biowest, Riverside, UK), 1% penicillin/Streptomycin and 1% L-Glutamine (Lonza, Basel, Switzerland.) at 37°C, 5% CO_2_. hAMSC were cultured using DMEM/F12 media supplemented with 5% platelets lysate (homemade) 1% penicillin/Streptomycin and 1% L-Glutamine using 75 cm^2^ flasks. Cell culture media was replaced twice a week and cells were subcultured once they reached 80-90% confluence. For subculturing, culture media was removed, washed using Ringer’s Lactate (Baxter, Deerfield, US), then 0.05% Trypsin/EDTA (Sigma Aldrich, St Louis, US) was added and incubated for three minutes, inactivated with complete media and cells were centrifugated, pelleted and cell viability determined by 0.4% Trypan Blue (Sigma Aldrich, St Louis, US).

**hAMSC Immunophenotype:**

hAMSC phenotype was tested by flow cytometry; 200 μl of cell suspension (1.5x10^5^ and 2x10^5^ hAMSC) were disposed into flow cytometry tubes. Then, 2 μl of antibody were added as following: positive markers CD73-APC, CD105-PE (Biolegend, San Diego, US) CD90-FITC, and CD44-FITC (Molecular Probes, Eugene, US) and negative markers CD34-PE, CD45-APC y HLA-DR-FITC (Biolegend, San Diego, US). Tubes were incubated for 30 minutes protected from light at room temperature. 10x10^3^ events per tube were recorded in a LSRFortessaTM Cell Analyzer (BD Biosciences, Franklin Lakes, US) and data was processed in FlowJo 10.7.1 (tableS1).

**Propidium Iodide incorporation:**

Cell membrane integrity was evaluated by flow cytometry by Propidium Iodide (PI 1μg/ml, InvitrogenTM, Waltham, US) incorporation obtaining the percentage of viability after direct treatment with plant extracts and the combination of plant extracts and hAMSC-CM. 1x10^5^ tumor cells were plated on 24 well plates using DMEM/F12 supplemented with 2.5% FBS and were allowed to attach overnight. Then, Anamu-SC or P2Et IC50, hAMSC-CM or combination were added for 24 and 48 hours; FBS free medium was used as control. Cells were recovered and resuspended in MEM without phenol red supplemented with 5% FBS and PI was added and incubated for 30 minutes at room temperature protected from light. At least 5000 events were capture in an LSRFortessaTM Cell Analyzer Flow cytometer (BD Biosciences, Franklin Lakes, US) and data was analyzed with FlowJo 10.7.1.

**Table S1) Immunophenotype of hAMSC.**

|  | | Marker | hAMSC 1 | hAMSC 3 | hAMSC4 |
| --- | --- | --- | --- | --- | --- |
|  | **+** | CD44 | 76.30% | 96.04% | 95.79% |
|  |  | CD73 | 99.98% | 99.95% | 99.96% |
|  |  | CD90 | 79.97% | 87.31% | 84.49% |
|  |  | CD105 | 98.40% | 99.57% | 99.10% |
|  | **-** | CD34 | 16.8% | 11% | 7.3% |
|  |  | CD45 | 6% | 14.2% | 2.63% |
|  |  | HLA-DR | 6.5% | 28.6% | 2.1% |

**Table S2) Primers used for RT qPCR.**

| Gene | Fw | Rv |
| --- | --- | --- |
| RANTES | CAGCACGTGGACCTCGCACA | GGCAGTGGGCGGGCAATGTA |
| IL-6 | AAGCCAGAGCTGTGCAGTGAGTA | TGTCCTGCAGCCACTGGTTC |
| TRAIL | TGTGTGGCTGTAACTTACGTGTACTTT | GGGCTGTTCATACTCTCTTCGTCAT |
| TGF-β1 | AGCGACTCGCCAGAGTGGTTA | GCAGTGTGTTATCCCTGCTGTCA |
| VEGF-A | ACTTCCCCAAATCACTGTGG | GTCACTCACTTTGCCCCTGT |

The thermal cycling conditions were denaturation step at 95°C for 30 seconds, 40 cycles of 15 seconds at 95°C, 60 seconds at annealing temperature, and 40 seconds at 72°C followed by a dissociation stage. The relative expression levels of each gene were normalized to housekeeping gene β2-Microglobulin. Relative expression was calculated using the 2^−ΔΔCT^ comparative method.
